# The relationship between short-video addiction tendency and inhibitory control in university students: ERP evidence from a Go/No-go task

**DOI:** 10.3389/fpsyg.2026.1824227

**Published:** 2026-06-25

**Authors:** Bingqi Li, Narina A. Samah, Tingting Liu

**Affiliations:** 1 Faculty of Educational Sciences and Technology, Universiti Teknologi Malaysia, Johor Bahru, Malaysia; 2 Faculty of Education, Universiti Malaya, Kuala Lumpur, Malaysia

**Keywords:** event-related potentials, inhibitory control, N2, P3, short-video addiction tendency, university students

## Abstract

**Objective:**

This study aimed to examine the relationship between short-video addiction tendency and inhibitory control performance among university students.

**Method:**

Based on the preset screening criteria of the Smartphone Addiction Inventory (SPAI) and the adapted Internet Addiction Test (IAT), candidate participants were screened, and 30 university students with higher short-video addiction tendency were finally included in the addiction group, while 31 university students were included in the control group. All participants completed a Go/No-go task, and behavioral indicators and event-related potential (ERP) data were collected simultaneously. Independent-samples t tests, Holm-Bonferroni correction for multiple comparisons, and repeated-measures analysis of variance were used to compare the differences between the two groups in reaction time, accuracy, false alarm rate, and the mean amplitudes of the N2 and P3 components.

**Results:**

At the behavioral level, the addiction group showed numerically lower accuracy and higher false alarm rate than the control group, but after Holm-Bonferroni correction, the group differences in behavioral indicators did not reach statistical significance. In the ERP results, the mean amplitude of the N2 component was significantly more negative in the control group than in the addiction group, while no significant group difference was found in the mean amplitude of P3.

**Conclusion:**

Individuals with short-video addiction tendency showed certain trends in behavioral inhibition-related indicators, but the behavioral evidence should still be interpreted with caution. The ERP results preliminarily suggest that short-video addiction tendency may be related to insufficient conflict monitoring or control initiation in the early stage of inhibitory control.

## 1 Introduction

Short-video platforms are characterized by rapid content updates, accurate recommendations, immediate feedback, and low barriers to use. These features may strengthen users’ tendency toward continuous use. Short-video addiction is usually manifested as a high level of dependence on short-video platforms and frequent use that is difficult to control (Yang et al., 2016). In addition to continuous attention to and repeated access to short-video content, related problematic use may also be accompanied by craving, increased use time, control difficulties, and continued use despite awareness of possible negative consequences. Previous studies have shown that excessive short-video use may be associated with decreased attention, time management difficulties, and affected academic performance (Chen et al., 2023; Tian et al., 2023; Ye et al., 2022). Therefore, short-video addiction tendency is not only reflected in a high frequency of use, but may also be related to individuals’ difficulty in controlling immediate reward cues (Li et al., 2025).

Inhibitory control is an important component of executive function. According to Diamond (2013), inhibitory control refers to the ability of individuals to control their attention, behavior, thoughts, and emotions in order to overcome strong internal tendencies or external temptations and perform more appropriate or needed behaviors. This ability plays an important role in suppressing dominant responses, reducing impulsive behaviors, and maintaining goal-directed behaviors (Diamond, 2013; Miyake and Friedman, 2012). In the context of short-video use, individuals need to inhibit the impulse to continue browsing, click repeatedly, or seek immediate gratification. Therefore, inhibitory control may be one of the important cognitive factors for understanding short-video addiction tendency.

The Go/No-go task is a commonly used experimental paradigm for assessing inhibitory control (Littman and Takács, 2017; Shields and Yonelinas, 2025). In this task, Go trials appear more frequently and easily form a dominant response, whereas No-go trials require individuals to inhibit the already formed key-press response (Hagen et al., 2016). Generally, a lower No-go false alarm rate reflects better response inhibition ability (Diamond, 2013). In event-related potential (ERP) studies, No-go stimuli usually elicit two components related to response inhibition processing, namely N2 and P3 (Guo et al., 2018). No-go N2 is usually associated with conflict monitoring and early cognitive control, and is often measured by the N2 amplitude elicited under the No-go condition or the N2 difference amplitude between the No-go and Go conditions (N2d). A larger No-go N2 or N2d amplitude may reflect stronger conflict monitoring ability (Cheng et al., 2017). No-go P3 is usually related to the later processing of response inhibition and motor inhibition, and is often measured by the No-go P3 amplitude or the P3 difference amplitude between the No-go and Go conditions (P3d). A larger No-go P3 or P3d amplitude may indicate stronger response inhibition ability (Enriquez-Geppert et al., 2010).

Existing studies on behavioral addiction provide indirect evidence for the relationship between short-video addiction tendency and inhibitory control. Dong et al. (2010) used a Go/No-go task combined with ERP indicators to examine inhibitory control in individuals with Internet addiction. They found that the Internet addiction group showed lower No-go N2 amplitude, together with higher No-go P3 amplitude and longer latency, suggesting that they may have insufficient early conflict detection and may need to invest more cognitive resources in the later stage to complete inhibition. Moretta and Buodo (2021) found that problematic social networking site users had lower Go and No-go accuracy than non-problematic users in an emotional Go/No-go task, and also showed lower No-go P3 amplitude. Gao et al. (2020) found that mobile phone application-related backgrounds increased error responses among problematic mobile phone users and were accompanied by weaker No-go P3 amplitude. These studies indicate that problematic use related to the Internet, social media, and mobile phones may be associated with weakened inhibitory control performance. However, ERP findings across different studies are not completely consistent, suggesting that this issue still needs to be further examined in specific platforms and specific use contexts.

As a problematic behavior related to the use of short-video platforms, short-video addiction tendency may share similar inhibitory control characteristics with Internet addiction, social media addiction, and problematic mobile phone use. However, ERP research on the relationship between short-video addiction tendency and inhibitory control is still relatively limited. In particular, it remains unclear whether individuals with short-video addiction tendency show differences in behavioral inhibition-related indicators in the Go/No-go task, and whether these differences are mainly reflected in the early conflict monitoring stage indexed by N2 or the later inhibitory processing stage indexed by P3. Therefore, this study used the Go/No-go task combined with ERP technology to examine the differences between the addiction group and the control group in behavioral indicators, including Go reaction time, No-go accuracy, and false alarm rate, as well as ERP components under the No-go condition, including the mean amplitudes of N2 and P3, in order to preliminarily explore the relationship between short-video addiction tendency and inhibitory control performance among university students.

## 2 Methods

### 2.1 Participants

This study distributed the Smartphone Addiction Inventory (SPAI) (Lin et al., 2014) and the adapted Internet Addiction Test (IAT) (Young, 1998) to 2,005 undergraduate students at Xizang University. A total of 1,930 valid questionnaires were collected, with an effective response rate of 96.25%. According to the preset screening criteria of the two scales, candidate participants who met the conditions for the addiction group and the control group were first screened. The number of eligible candidate participants was larger than the final included sample. Considering the long duration of EEG data collection, the high requirements of the experimental procedure, and the need to ensure that participants could complete the full Go/No-go task and EEG recording, the researchers contacted eligible students based on the candidate list and invited them to participate in the EEG experiment. Finally, among the candidate participants who volunteered to participate and were able to complete the full experimental procedure, 30 participants with higher short-video addiction tendency were included in the addiction group, including 25 males, with both SPAI and adapted IAT scores above 40. In addition, 31 participants with lower short-video addiction tendency were included in the control group, including 27 males, with both SPAI and adapted IAT scores below 40.

All participants were Han Chinese, with a mean age of 20.39 ± 1.56 years. They had normal or corrected-to-normal vision and were all right-handed. All participants were physically healthy and had no dependence on substances such as nicotine or caffeine. This study was approved by the Ethics Committee of Xizang University. Before the experiment, all participants signed informed consent forms. After the experiment, all participants received corresponding compensation.

### 2.2 Stimuli and procedure

#### 2.2.1 Go/No-go task

**Figure 1:**
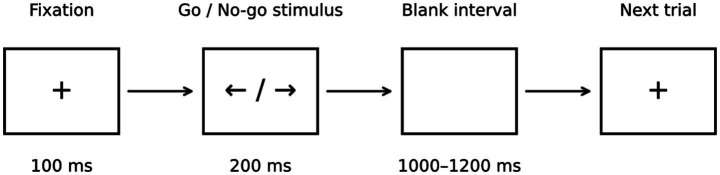
Stimuli and experiment procedure of this study.

This experiment adopted a Go/No-go task to measure participants’ inhibitory control function (Zhang et al., 2014). The task was completed on a computer using E-Prime 2.0, with a viewing distance of approximately 60 cm. The stimuli were left-pointing arrows (Go) and right-pointing arrows (No-go). The procedure was as follows: participants were required to make a keypress response to Go trials and ignore No-go trials. Each stimulus sequence first presented a fixation cross (“+”) at the center of the screen for 100 ms, followed by the stimulus presented for 200 ms. The interval between each stimulus and the next fixation cross was randomly varied within a range of 1,000–1,200 ms, and the random interval was used to avoid contamination of the data by expectancy effects. The experiment consisted of 400 randomized trials (160 No-go and 240 Go). All stimuli were presented in random order to participants, and they were instructed to trade off speed and accuracy. To ensure that participants correctly understood the task and to rule out the influence of practice effects, participants were required to achieve a hit rate of 98% or higher during the practice phase (Figure 1).

#### 2.2.2 Adapted internet addiction test (IAT)

This study used the Chinese version of Young’s (1998) 20-item IAT, and replaced “Internet” in the items with “short-video APP” according to the research context. The questionnaire contains 20 items and uses a 5-point scoring system, where 1 indicates “rarely” and 5 indicates “always.” A higher score indicates more serious problems related to short-video APP use. This questionnaire was mainly used to screen problematic use of short-video APPs, including salience, excessive use, control difficulties, effects on study or life responsibilities, effects on social life, and anticipatory use. In this study, the Cronbach’s *α* of this scale was 0.861. It should be noted that the adapted IAT was only used as a screening tool for short-video use problems, and was not a specialized clinical diagnostic scale for short-video addiction.

#### 2.2.3 Smartphone addiction inventory (SPAI)

The Smartphone Addiction Inventory was developed by Lin et al. (2014) and contains 26 items, including four factors: compulsive behavior, withdrawal, tolerance, and functional impairment. The questionnaire uses a 4-point scoring system, where 1 indicates “strongly disagree” and 4 indicates “strongly agree.” The total score is obtained by summing the scores of all items. A higher score indicates a more obvious addiction tendency related to smartphone use. In this study, the Cronbach’s *α* of this scale was 0.875. The SPAI was mainly used in this study to assist in screening smartphone use-related addiction tendency, in order to improve the strictness of selecting the addiction tendency group.

### 2.3 ERP recording

Electroencephalography (EEG) was recorded using the NeuronScan ERP recording and analysis system with a 64-channel electrode cap extended according to the international 10–20 system. The REF electrode was used as the reference electrode. Electrodes were placed at the outer canthi of both eyes to record horizontal electrooculogram (HEOG), and electrodes were placed above and below the left eye to record vertical electrooculogram (VEOG). The sampling rate was 500 Hz, and the online filter was set at 0.05–100 Hz. The impedance of all scalp electrodes was kept below 5 kΩ.

Offline processing was completed in MATLAB (R2018b; MathWorks, Inc., MA, USA) using the EEGLAB toolbox (Delorme and Makeig, 2004) and the ERPLAB plugin (Lopez-Calderon and Luck, 2014). During offline analysis, the data were re-referenced to the average of the bilateral mastoids (M1 and M2), and continuous EEG data were filtered with an offline band-pass filter of 0.1–30 Hz. Independent component analysis (ICA) was then performed in EEGLAB, and eye movement/blink-related artifact components were manually identified and removed based on the scalp distribution and time-course characteristics of the components. ERP data were segmented from −200 to 1,000 ms relative to stimulus onset, and baseline correction was performed using the −200 to 0 ms interval. Automatic artifact rejection was conducted using ERPLAB, and trials with amplitudes exceeding ±100 μV in any channel were removed. Finally, only trials with correct responses were averaged for ERP analysis.

Based on the typical scalp distribution of ERP components related to inhibitory control in the Go/No-go task, this study selected nine electrode sites for analysis, including the frontal region (F1, Fz, F2), frontocentral region (FC1, FCz, FC2), and central region (C1, Cz, C2). Previous studies have shown that No-go N2 usually appears approximately 200–350 ms after stimulus onset and is mainly distributed over the frontal and frontocentral regions, reflecting conflict monitoring or early inhibitory control processing (Folstein and Van Petten, 2008; Nieuwenhuis et al., 2003). No-go P3 usually appears approximately 300–600 ms after stimulus onset and is mainly distributed over the frontocentral and central regions, and is related to response inhibition and post-inhibition evaluation processing (Polich, 2007; Smith et al., 2008). Therefore, based on previous studies and the time ranges in which the N2 and P3 components appeared in the grand-average waveforms of this study, the mean amplitude time window for N2 was set at 200–350 ms, and the mean amplitude time window for P3 was set at 350–550 ms. The same time windows were used across all groups and conditions to avoid analytical bias caused by data-driven time-window selection.

### 2.4 Data analysis and statistical processing

Behavioral and EEG data were analyzed using SPSS 23.0 software. Independent-samples t tests were conducted between the two groups for the hit rate and reaction time under the Go condition and the false alarm rate under the No-go condition in the Go/No-go task. To control the inflation of Type I error that may be caused by multiple comparisons of behavioral indicators, the Holm-Bonferroni method was used to correct the group comparisons of the three behavioral indicators. Repeated-measures analysis of variance was conducted on the mean amplitudes of N2 and P3 with group (addiction group, control group) × electrode position (frontal region, frontocentral region, central region). For *p* values that did not meet the assumption of sphericity, Greenhouse–Geisser correction was applied. When the main effect of electrode position was significant, Bonferroni correction was further used for *post hoc* pairwise comparisons. All statistical tests were two-tailed, and the significance level was set at *α* = 0.05.

## 3 Results

### 3.1 Behavioral data

Independent-samples t tests were conducted on reaction time, accuracy, and false alarm rate between the two groups, and the Holm-Bonferroni method was used to correct the group comparisons of the three behavioral indicators for multiple comparisons. The uncorrected results showed that the addiction group had numerically lower accuracy than the control group, *t*(59) = −2.199, *p* = 0.032; the false alarm rate was numerically higher than that of the control group, *t*(59) = 2.362, *p* = 0.022; and there was no significant difference between the two groups in reaction time. After Holm-Bonferroni correction, the group differences in accuracy and false alarm rate did not reach statistical significance. Therefore, the behavioral results only suggest that the short-video addiction group may show a trend of lower accuracy and higher false alarm rate (Table 1).

**Table 1:** Comparison of behavioral data between the addiction group and the control group (M ± SD).

Behavioral indicators	Group		*t*	*p*
	Addiction group	Control group		
reaction time	354.590 ± 32.391	357.623 ± 28.769	−0.387	0.700
accuracy	0.862 ± 0.064	0.893 ± 0.046	−2.199	0.032
False alarm rate	0.113 ± 0.076	0.076 ± 0.042	2.362	0.022

The *p* values shown in the table are uncorrected *p* values. After Holm-Bonferroni correction, the group differences in accuracy and false alarm rate did not reach statistical significance.

### 3.2 ERP data

#### 3.2.1 Nogo-N2

**Figure 2:**
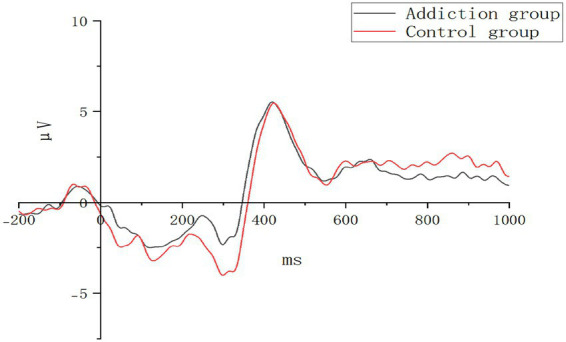
ERP waveform of the addiction and control groups.

**Figure 3:**
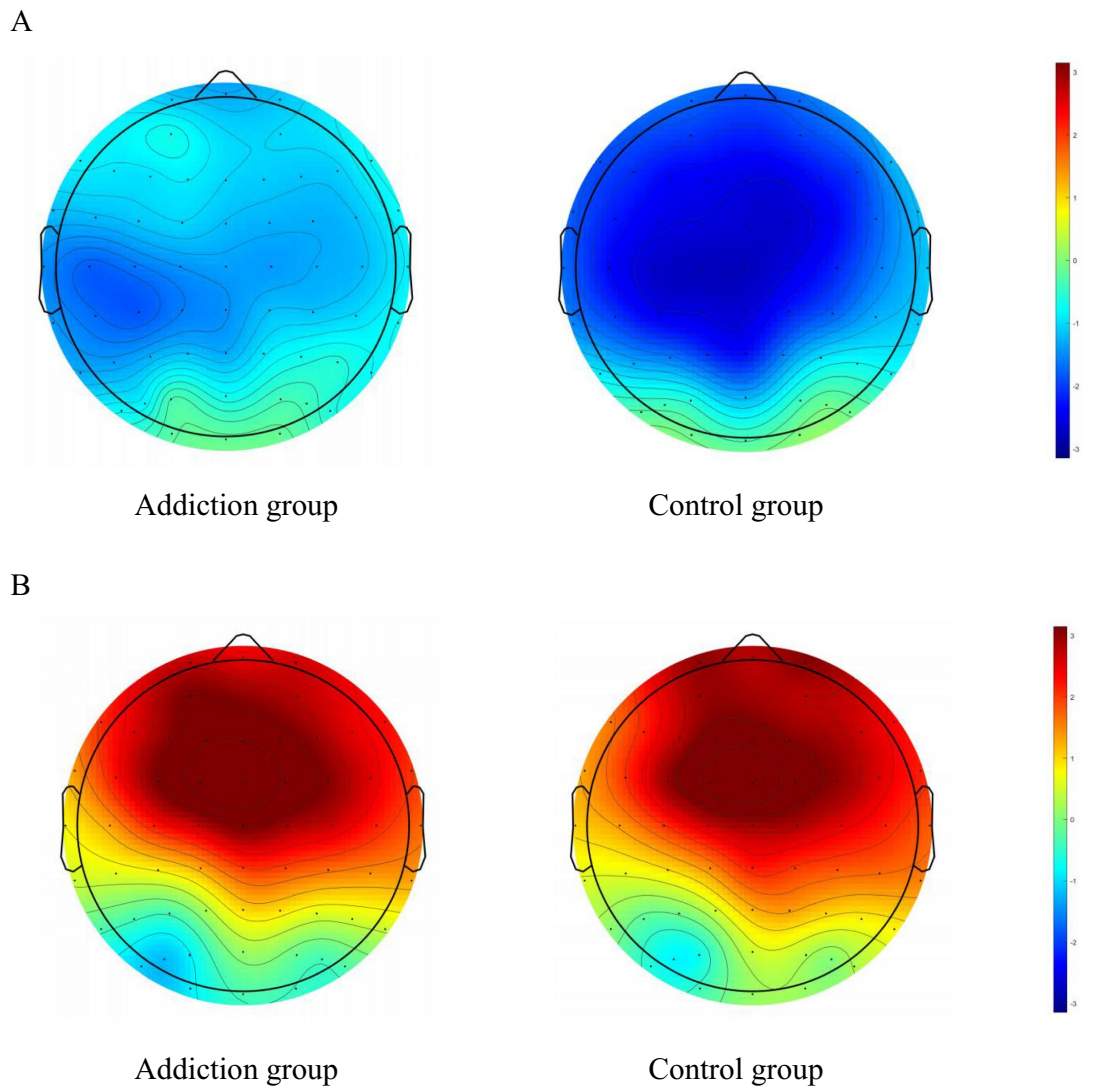
ERP topography of the addiction and control groups. **(A)** N2 (200–350ms). **(B)** P3 (350–550ms).

The mean amplitude of the Nogo-N2 component was analyzed within the 200–350 ms time window. A two-way repeated-measures analysis of variance was conducted on the mean amplitude of Nogo-N2 with 2 (group: addiction group, control group) × 3 (electrode position: frontal region, frontocentral region, central region). The results showed that the main effect of group was significant, *F*(1, 59) = 4.662, *p* = 0.035, partial η^2^ = 0.073, and the mean N2 amplitude of the control group was significantly more negative than that of the addiction group. The main effect of electrode position was not significant, and the interaction between group and electrode position was also not significant (Table 2; Figures 2, 3).

**Table 2:** Mean amplitudes of No-go N2 and No-go P3 in the addiction group and the control group (M ± SD).

Mean amplitude	Addiction group	Control group
Nogo-N2		
Frontal	−1.016 ± 2.846	−2.527 ± 2.199
Frontocentral	−1.168 ± 2.989	−2.638 ± 2.369
Central	−1.408 ± 2.813	−2.699 ± 2.554
Nogo-P3		
Frontal	3.543 ± 2.235	3.287 ± 2.213
Frontocentral	3.806 ± 2.365	3.617 ± 2.352
Central	2.960 ± 2.285	2.806 ± 2.237

#### 3.2.2 Nogo-P3

The mean amplitude of the Nogo-P3 component was analyzed within the 350–550 ms time window. A two-way repeated-measures analysis of variance was conducted on the mean amplitude of Nogo-P3 with 2 (group: addiction group, control group) × 3 (electrode position: frontal region, frontocentral region, central region). The results showed that the main effect of group was not significant; the main effect of electrode position was significant, *F*(2, 118) = 22.837, *p* < 0.001, partial η^2^ = 0.279. Further *post hoc* comparisons using Bonferroni correction showed that the mean amplitudes in the frontal and frontocentral regions were significantly greater than those in the central region. The interaction between electrode position and group was not significant (Table 2; Figures 2, 3).

## 4 Discussion

This study used the Go/No-go task combined with event-related potential (ERP) technology to examine the relationship between short-video addiction tendency and inhibitory control performance among university students. The results showed that the addiction group showed certain trends of decreased accuracy and increased false alarm rate, but these trends did not reach statistical significance after Holm-Bonferroni correction for multiple comparisons. In the ERP results, the mean amplitude of the N2 component was significantly more negative in the control group than in the addiction group. This result suggests that the addiction group may have certain insufficiency in the early stage of conflict monitoring or inhibitory control initiation.

From the behavioral results, the addiction group had numerically lower accuracy and higher false alarm rate than the control group, but these differences did not reach statistical significance after multiple comparison correction. Therefore, the behavioral results should be interpreted with caution and cannot be used alone as direct evidence of significantly impaired inhibitory control in the addiction group. Existing studies on behavioral addiction suggest that a higher No-go false alarm rate or lower task accuracy is usually associated with weakened inhibitory control (Fathi et al., 2024; Jacquet et al., 2023). The behavioral results of this study are generally consistent with these findings in direction, but the statistical evidence remains weak. Future studies need to further verify this issue with larger samples and more strictly controlled conditions.

In the Go/No-go task, No-go N2 is often regarded as an indicator reflecting early cognitive control demands, and is related to conflict monitoring and the early mobilization of inhibitory control (Falkenstein et al., 1999; Folstein and Van Petten, 2008; Eimer, 1993). This study found that the mean N2 amplitude of the control group was significantly more negative than that of the addiction group. This result suggests that, compared with the control group, the addiction group may have more difficulty in effectively detecting response conflict and mobilizing early inhibitory control resources in time under the No-go condition. This finding is generally consistent with previous evidence showing weaker No-go N2 in individuals with problematic Internet use during inhibition tasks (Luijten et al., 2014; Zhou et al., 2010). Combined with the direction of the behavioral results, the weaker N2 amplitude in the addiction group may be related to its trend of lower accuracy and higher false alarm rate. However, since the behavioral differences did not reach statistical significance after multiple comparison correction, this interpretation should still be treated with caution.

No-go P3 is a late positive wave elicited by No-go stimuli in the Go/No-go task, and is usually considered to reflect the later processing of inhibitory control, such as inhibition outcome evaluation and task-related information updating (Smith et al., 2008; Wessel and Aron, 2015; Xia et al., 2020). This study did not find a significant difference between the two groups in the mean P3 amplitude. This indicates that this study did not obtain evidence of obvious abnormalities in the later inhibition evaluation and updating stage in the addiction group. Compared with the P3 results, the N2 results are more likely to suggest that the inhibitory control-related differences in the addiction group may mainly appear in the early control process, especially in the stages of response conflict monitoring and early recruitment of inhibitory control (Kim et al., 2017).

This study still has several limitations. First, the addiction group in this study was a group with short-video addiction tendency screened based on the SPAI and the adapted IAT, rather than short-video addicts in the clinical diagnostic sense. Since the adapted IAT was only used to screen problematic use of short-video APPs, and the SPAI mainly reflects smartphone use-related addiction tendency, this study cannot completely exclude the influence of general smartphone use problems or Internet use problems on the results. Therefore, the conclusions of this study should be cautiously interpreted as possible differences in inhibitory control-related indicators among individuals with short-video addiction tendency. Second, the group differences in behavioral indicators did not reach statistical significance after multiple comparison correction. Therefore, the interpretation of behavioral inhibitory control performance in the addiction group should be regarded as preliminary evidence. Third, the analysis of reaction time data in this study was mainly based on the mean reaction time of each participant, and trial-level linear mixed models or ex-Gaussian distribution models were not further used to analyze the distribution characteristics of reaction time. Since no significant group difference was found in reaction time in this study, reaction time was not used as the main evidence for explaining differences in inhibitory control. Fourth, this study used neutral arrow stimuli to complete the Go/No-go task, which mainly reflects general response inhibition ability and cannot directly explain inhibitory control characteristics under short-video-related cues. Fifth, this study mainly analyzed the mean amplitudes of N2 and P3 under the No-go condition, and did not further calculate the No-go minus Go difference waves. Therefore, it is difficult to completely exclude the influence of general cognitive processing differences. Finally, there was a gender imbalance in the sample of this study, with more male participants. Therefore, caution is needed when generalizing the findings to female populations.

Future studies can be further improved in the following aspects. First, specially validated short-video addiction scales can be used, together with interviews, actual use duration, or platform use records, to improve the construct validity of grouping. Second, the sample size can be expanded and the gender ratio can be balanced to enhance the stability and generalizability of the findings. Third, trial-level linear mixed models or ex-Gaussian distribution models can be used to analyze the distribution characteristics of reaction time in more detail. Fourth, short-video-related stimuli or cue-specific Go/No-go tasks can be used to further examine inhibitory control performance among individuals with addiction tendency under short-video-related cues. Fifth, No-go minus Go difference waves can be further calculated to more clearly separate ERP activities related to response inhibition, and fMRI and other techniques can be combined to further explore the neural mechanisms between short-video addiction tendency and inhibitory control.

## 5 Conclusion

In summary, this study found that the addiction group showed certain trends of decreased accuracy and increased false alarm rate, but the behavioral differences did not reach statistical significance after multiple comparison correction. The ERP results showed that the mean N2 amplitude of the control group was significantly more negative than that of the addiction group, while no significant group difference was found in the mean P3 amplitude. This result preliminarily suggests that short-video addiction tendency may be related to insufficient conflict monitoring or control initiation in the early stage of inhibitory control. This study provides preliminary neuroelectrophysiological evidence for understanding the relationship between short-video addiction tendency and inhibitory control.

## Data Availability

The raw data supporting the conclusions of this article will be made available by the authors, without undue reservation.
